# Increasing Pre-pregnancy Body Mass Index and Pregnancy Outcomes in the United States

**DOI:** 10.7759/cureus.28695

**Published:** 2022-09-02

**Authors:** Oluwasegun A Akinyemi, Resham Tanna, Stella Adetokunbo, Ofure Omokhodion, Mojisola Fasokun, Akinwale S Akingbule, Chidi Martins, Mary Fakorede, Temitayo Ogundipe, Oladunni Filani

**Affiliations:** 1 Department of Health Policy and Management, University of Maryland School of Public Health, College Park, USA; 2 Department of Surgery, Howard University, Washington DC, USA; 3 Department of Obstetrics and Gynecology, Spartan Health Sciences University, Vieux Fort, LCA; 4 Department of Internal Medicine, The Einstein Medical Center, Philadelphia, USA; 5 Department of Obstetrics and Gynecology, University College Hospital, Ibadan, NGA; 6 Department of Epidemiology and Public Health, University of Alabama at Birmingham, Birmingham, USA; 7 Department of General Practice, Primary Care, and Public Health, Ozark Valley Medical Clinic, Branson, USA; 8 Department of Obstetrics and Gynecology, Howard University College of Medicine, Washington DC, USA; 9 Department of Addiction Medicine, Johns Hopkins University School of Medicine, Baltimore, USA; 10 Department of Community and Family Medicine, Howard University Hospital, Washington DC, USA

**Keywords:** perinatal outcomes, macrosomia, cesarean section, gestational weight gain, classes of obesity, obesity prevention, pregnancy outcomes, pre-pregnancy body mass index

## Abstract

Introduction: As many Americans are becoming overweight or obese, increased body mass index (BMI) is fast becoming normalized. There is a need for more research that highlights the association between pre-pregnancy obesity and adverse pregnancy outcomes.

Aim: To determine the association between increasing pre-pregnancy BMI and adverse pregnancy outcomes.

Methods: We utilized the United States Vital Statistics records to collate data on all childbirths in the United States between 2015 and 2019. We determined the association between increasing pre-pregnancy BMI and adverse pregnancy outcomes using multivariate analysis. Neonatal outcomes measures include the five-minute Apgar score, neonatal unit admission, neonates receiving assisted ventilation > six hours, neonatal antibiotics use, and neonatal seizures. Maternal outcomes include cesarean section rate, mothers requiring blood transfusion, unplanned hysterectomy, and intensive care unit admission. In addition, we controlled for maternal parameters such as race/ethnicity, age, insurance type, and pre-existing conditions such as chronic hypertension and prediabetes. Other covariates include paternal race, age and education level, gestational diabetes mellitus, induction of labor, weight gain during pregnancy, gestational age at delivery, and delivery weight.

Results: We studied 15,627,572 deliveries in the US Vital Statistics records between 2015 and 2019. Among these women, 3.36% were underweight, 43.19% were with a normal BMI, 26.34% were overweight, 14.73% were in the obese class I, 7.23% were in the obese class II, and 5.14% were in the obese class III. Increasing pre-pregnancy BMI was associated with significant adverse outcomes across all measures of maternal and neonatal outcomes.

Conclusion: A strong association exists between increasing pre-pregnancy BMI and adverse maternal and neonatal outcomes. The higher risk of adverse pregnancy outcomes among overweight and obese women remained even after controlling for other traditional risk factors of adverse maternal and neonatal outcomes.

## Introduction

Overweight and obesity are a state of excess or abnormal fat accumulation that may impair health. The World Health Organization (WHO) defines overweight as a body mass index (BMI) > 25-29.9 kg/m^2^ and obesity as BMI > 30kg/m^2^. In 2016, over 1.9 billion adults were overweight globally, with 650 million of this population being obese [1]. Globally, the number of obese individuals has nearly tripled since 1975 [2]. In 2008, the annual cost of obesity to the US healthcare system was around $147 billion annually [3]. This increased to $260.6 billion in 2016 [4]. Obesity is estimated to account for productivity loss of up to about $13.4 to $26.8 billion or $271 to $542 per employee with obesity [5].

Globally, 40% of women were overweight in 2016, and 15% of women were obese in the same year [6]. In the United States, 27.5% of women were overweight, 41.9% were obese, and 11.5% were severely obese, according to the 2017-2018 National Health and Nutrition Examination Survey (NHANES) data, with 56.9% of non-Hispanic Black women being obese [7]. The annual individual cost for obesity-related treatment was $4,879 for women in 2001 [8].

Maternal obesity is associated with an increased risk of type 2 diabetes, hypertension, and cardiovascular disease. In addition, obese women have an increased risk for pregnancy complications such as gestational diabetes, preeclampsia, labor induction, cesarean section, and wound infection. Furthermore, their fetuses risk macrosomia and fetal distress [9].

Healthy People 2020 reported that maintaining a healthy body weight can reduce complications in obese women during pregnancy. The Healthy People 2020's obesity target was 30.5% from a baseline of 33.9% among adults aged 20 years and above in 2005-2008. However, the obesity rate increased to 38.6% in 2013-2016 [10]. Hutchesson et al. concluded that providing an efficient behavioral intervention focusing on improved health behavior and weight management to young adult females could improve future outcomes for both planned and unplanned pregnancies [11].

This study aims to investigate the association between pre-pregnancy BMI and adverse pregnancy outcomes. Reducing the incidence of adverse pregnancy outcomes requires intervention directed toward obese and overweight women before they conceive.

This article was previously posted to the Research Square preprint server on August 4, 2022.

## Materials and methods

Study design and data sources

We extracted the data for this study from the US Vital Statistics Records 2015-2019. The National Vital Statistics Records database provides complete information regarding births and deaths in the United States [12].

We explored the association between different classes of obesity and nine measures of maternal and neonatal pregnancy outcomes. Since the database is de-identified and publicly available, ethical clearance or Institutional Review Board approval was unnecessary.

Study population

We studied 15,627,572 deliveries recorded in the United States Vital Statistics records between January 2015 and December 2019. The study cohort included women of all races/ethnicities. However, women with missing vital information such as maternal race/ethnicity, BMI, age, and measured pregnancy outcomes were excluded from the study.

Patient characteristics and risk factors

Covariates utilized in this study were identified based on existing literature, including maternal and paternal demographics and obstetric and medical history. These factors included maternal race/ethnicity defined as non-Hispanic White (White), non-Hispanic Black (Black), Hispanic, and non-Hispanic other (other), maternal insurance types (private insurance, public insurance (Medicaid), and uninsured), and maternal education level defined as either high school, college, or advanced education. Other factors included in the study are maternal age, paternal race/ethnicity, paternal age, paternal education level, pre-pregnancy diabetes, hypertension, pre-pregnancy obesity, cesarean section and previous cesarean section (PCS), augmentation and labor induction, and delivery weight.

Definition of study outcomes

There were nine primary measures of pregnancy outcome. The maternal parameters were cesarean section, mothers requiring blood transfusion during labor or delivery (maternal blood transfusion), unplanned hysterectomy, and maternal admission to the intensive care unit (maternal ICU admissions). The neonatal outcomes included a low fifth-minute Apgar score (defined as Apgar score < 7), neonatal intensive care unit (NICU) admissions, neonates receiving assisted ventilation for ≥ six hours after delivery, neonatal antibiotics use, and neonatal seizures.

Statistical analysis

We expressed categorical variables as frequencies and percentages and continuous variables as mean ± standard deviations. Chi-square and independent sample t-tests were utilized to compare categorical and continuous variables. Using chi-square, baseline characteristics were stratified across the different classes of pre-pregnancy BMI (underweight, normal, obesity class I, obesity class II, and obesity class III). With a significant association, a post hoc analysis was performed between all groups to define the source of statistical significance. Unadjusted odds ratios (ORs) and 95% CIs for the association of study variables with pregnancy outcomes were calculated from bivariate logistic models.

We adjusted for maternal age, maternal race/ethnicity, maternal education, maternal insurance types, paternal race/ethnicity, paternal age, paternal education level, pre-pregnancy diabetes, chronic hypertension, weight gain in pregnancy, PCS, induction of labor, delivery weight, and gestational age at delivery in our final multivariate analysis. We then determined the association between different classes of pre-pregnancy BMI and maternal and neonatal pregnancy outcomes. A two-tailed p-value < 0.05 was regarded as statistically significant. All statistical analyses were performed using STATA 16 (StataCorp, College Station, TX).

## Results

We conducted a retrospective analysis of 15,627,572 deliveries. Of the women, 3.4% were underweight with a BMI < 18.5, 43.2% were with a normal pre-pregnancy BMI (18.5-24.9), and 26.3% were overweight (25.0-29.9), with 14.7% in the obesity class I group (30.0-34.9), 7.2% with obesity class II (BMI 35-39.9), and 5.1% with obesity class III.

Table 1 highlights the baseline distribution of studied variables stratified by maternal pre-pregnancy BMI categories. Most of the women were between the ages of 20 and 34. Among Black women, the proportion of women in each category of pre-pregnancy BMI group increases as the pre-pregnancy BMI increases: underweight (13.2%), normal pre-pregnancy BMI (10.82%), overweight (14.48%), obesity class I (17.66%), obesity class II (19.91%), and obesity class III (24.37%).

**Table 1:** Socio-demographic parameters and risk factors stratified by classes of body mass index CS: cesarean section; GDM: gestational diabetes mellitus.

Variables	Underweight (n = 509,596)	Normal (n = 6,541,345)	Overweight (n = 3,990,093)	Obese class I (n = 2,231,607)	Obese class II (n = 1,094,687)	Obese class III (n = 778,725)	P-value
Mother's age							<0.001
<19 years	11.28%	6.09%	4.61%	4.00%	3.43%	2.56%	
20-34 years	78.02%	76.86%	76.92%	77.76%	78.98%	80.04%	
35-49 years	10.69%	17.03%	18.44%	18.21%	17.58%	17.39%	
≥50 years	0.01%	0.02%	0.03%	0.02%	0.02%	0.01%	
Race/ethnicity							<0.001
White	51.04%	56.53%	49.37%	47.02%	48.80%	49.05%	
Blacks	13.12%	10.82%	14.48%	17.66%	19.91%	24.37%	
Hispanics	18.83%	20.85%	27.62%	28.60%	25.72%	21.71%	
Others	17.02%	11.80%	8.53%	6.71%	5.57%	4.87%	
Maternal education							<0.001
High school	47.16%	34.51%	39.43%	43.10%	43.66%	44.42%	
College	42.38%	49.74%	49.41%	48.93%	49.75%	50.17%	
Advanced	10.46%	15.75%	11.16%	7.96%	6.59%	5.41%	
Insurance							<0.001
Private	40.32%	54.00%	48.76%	44.85%	43.78%	41.32%	
Medicaid	50.49%	37.23%	42.77%	47.81%	50.21%	53.87%	
Uninsured	5.62%	4.86%	4.27%	3.52%	2.63%	1.88%	
Others	3.57%	3.91%	4.20%	3.82%	3.37%	2.93%	
Father's race							<0.001
White	52.85%	58.67%	50.31%	47.24%	48.56%	48.59%	
Blacks	11.38%	9.66%	13.87%	17.25%	19.60%	23.98%	
Hispanics	18.87%	20.47%	27.64%	29.20%	26.69%	22.97%	
Others	16.90%	11.20%	8.19%	6.32%	5.15%	4.46%	
Father's age							<0.001
<45years	96.37%	96.26%	95.96%	95.88%	96.03%	96.01%	
45-65 years	3.59%	3.71%	4.01%	4.09%	3.94%	3.96%	
>65 year	0.04%	0.03%	0.03%	0.03%	0.02%	0.02%	
Father's education							<0.001
High school	45.20%	35.67%	44.31%	51.14%	54.30%	57.41%	
College	41.76%	48.84%	46.14%	42.77%	41.30%	39.39%	
Advanced	13.04%	15.49%	9.55%	6.08%	4.40%	3.20%	
Smoking	11.57%	6.30%	6.20%	7.12%	7.94%	8.45%	<0.001
Induction	21.98%	24.03%	26.60%	28.76%	30.77%	32.17%	<0.001
CS rate	21.67%	25.90%	32.41%	38.11%	43.81%	52.56%	<0.001
Prediabetes	0.25%	0.38%	0.78%	1.36%	2.08%	3.21%	<0.001
Chronic hypertension	0.43%	0.69%	1.52%	2.79%	4.59%	8.57%	<0.001
GDM	2.94%	3.75%	6.30%	9.15%	11.57%	14.25%	<0.001
Weight gain in pregnancy							<0.001
Low	1.80%	2.92%	8.07%	15.57%	24.43%	33.65%	
Normal	75.70%	73.98%	70.28%	68.17%	63.53%	56.78%	
Excessive	22.49%	23.10%	21.64%	16.26%	12.03%	9.56%	
Birth weight							<0.001
<2500	12.56%	7.91%	7.54%	8.08%	8.51%	9.27%	
2500-3999	84.77%	86.04%	84.00%	82.20%	80.51%	78.72%	
4000-4499	2.46%	5.42%	7.37%	8.25%	9.07%	9.59%	
>4500	0.21%	0.63%	1.09%	1.47%	1.91%	2.42%	
Gestational age							<0.001
<37 weeks	13.09%	10.56%	11.29%	12.46%	13.33%	14.67%	
37-39 weeks	56.37%	55.51%	56.11%	56.43%	56.77%	57.59%	
40 weeks	30.53%	33.93%	32.60%	31.11%	29.90%	27.73%	

There was a significant association between maternal age, race/ethnicity, maternal education, insurance type, and the different classes of pre-pregnancy BMI. Among women with normal pre-pregnancy BMI, 6.09% were less than 19 years, 76.86% were between 20 and 34 years, 17.03% were between 35 and 49 years, and 0.02% were more than 50 years. However, among women with obesity class III pre-pregnancy BMI, about 2.56% were below 19 years, 80.04% were between 20 and 34 years, 17.39% were between 35 and 49 years, and 0.01% were above the age of 50 years.

Among women with normal pre-pregnancy BMI, 56.53% were Whites, 10.82% were Blacks, 20.85% were Hispanics, and 11.80% belonged to other races. However, among women with class III obesity, 49.05% were Whites, 24.37% were Blacks, and 21.71% were Hispanics. While the proportion of Whites and other races (mainly Asian/Pacific Islanders and Native Americans) reduces as the BMI increases, the proportion of Blacks and Hispanics increases dramatically with increasing pre-pregnancy BMI. Considering the population distribution in the United States, according to the latest census data, Whites were underrepresented in the obese group while Blacks were overrepresented.

Among women with normal pre-pregnancy BMI, 34.51% had high school education as their highest level of education, 49.74% had a college education, and 15.75% had advanced education. Among the women with morbid obesity, 44.4% had a high school education as their highest level of education, 50.2% had college degrees, and only 5.4% had advanced degrees. As the BMI increases, there seems to be a sharp increase in the proportion of women with high school education, while there was a drastic reduction in the proportion of women with an advanced degree.

Among the women with normal pre-pregnancy BMI, 54.00% had private insurance, 37.23% utilized Medicaid, and 4.86% were uninsured. However, among women with class III obesity, 41.32% had private insurance, 53.87% had Medicaid, and 1.88% were uninsured. As the BMI increased, there was an increase in the proportion of women with Medicaid insurance. At the same time, there was a corresponding reduction in the proportion of women with private insurance or those who were uninsured.

The prevalence of comorbidities such as prediabetes, chronic hypertension, and incidence of gestational diabetes mellitus increases across the different classes of BMI.

Table 2 is a multivariate analysis showing the association between the different classes of BMI and different measures of adverse maternal outcomes. With increasing pre-pregnancy BMI, there was a corresponding increase in the risk of adverse maternal outcomes. The only exception was women who had a maternal blood transfusion during labor and delivery. Among these women with increasing BMI, there was an inverse association between the increasing BMI and the risk of adverse outcomes.

**Table 2:** Association between classes of BMI and maternal outcomes Model adjusted for maternal age, maternal race/ethnicity, maternal education, maternal insurance, paternal race/ethnicity, paternal age, paternal education level, pre-pregnancy diabetes, chronic hypertension, weight gain in pregnancy, previous cesarean section, induction of labor, delivery weight, and gestational age at delivery.

	Cesarean section rate	Maternal blood transfusion	Unplanned hysterectomy	Maternal ICU admissions	
Normal BMI	Reference				
Underweight	0.79 (0.78-0.80)	1.05 (1.00-1.11)	0.92 (0.78-1.09)	1.00 (0.92-1.09)	
Overweight	1.39 (1.38-1.39)	0.97 (0.95-0.99)	1.12 (1.05-1.20)	1.02 (0.98-1.06)	
Obese I	1.79 (1.78-1.79)	0.95 (0.93-0.98)	1.10 (1.02-1.19)	1.05 (1.00-1.10)	
Obese II	2.27 (2.26-2.28)	0.90 (0.87-0.93)	1.11 (1.00-1.24)	1.11 (1.05-1.17)	
Obese III	3.19 (3.17-3.21)	0.94 (0.90-0.98)	1.25 (1.12-1.41)	1.29 (1.21-1.37)	

Table 3 is a multivariate analysis that shows the association between the different classes of pre-pregnancy BMI and the risk of adverse neonatal outcomes. With an increase in maternal pre-pregnancy BMI, there is a corresponding increase in the risk of adverse neonatal outcomes. Models were adjusted for maternal and paternal parameters, demographics, and obstetric and medical history.

**Table 3:** Association between classes of BMI and neonatal outcomes Model adjusted for maternal age, maternal race/ethnicity, maternal education, maternal insurance, paternal race/ethnicity, paternal age, paternal education level, pre-pregnancy diabetes, chronic hypertension, weight gain in pregnancy, previous cesarean section, induction of labor, delivery weight, and gestational age at delivery. SCBU: special care baby unit.

	Low Apgar score	Assisted ventilation	SCBU admission	Neonatal antibiotics	Neonatal seizures
Normal BMI	Reference				
Underweight	0.88 (0.85-0.90)	0.86 (0.84-0.89)	0.90 (0.89-0.91)	0.94 (0.92-0.96)	0.88 (0.72-1.07)
Overweight	1.13 (1.12-1.15)	1.13 (1.12-1.15)	1.10 (1.10-1.11)	1.06 (1.05-1.07)	1.14 (1.05-1.23)
Obese I	1.35 (1.24-1.27)	1.25 (1.24-1.27)	1.19 (1.18-1.20)	1.07 (1.06-1.09)	1.23 (1.12-1.35)
Obese II	1.38 (1.36-1.41)	1.38 (1.36-1.41)	1.27 (1.26-1.28)	1.11 (1.09-1.13)	1.35 (1.20-1.52)
Obese III	1.63 (1.61-1.66)	1.55 (1.52-1.58)	1.44 (1.42-1.45)	1.16 (1.14-1.18)	1.20 (1.05-1.38)

## Discussion

Our main finding in this study was that increasing pre-pregnancy BMI is strongly associated with adverse maternal and perinatal outcomes. This association remained significant even after adjusting for independent risk factors for adverse pregnancy outcomes. This is in keeping with a meta-analysis of 13 studies that showed a linear relationship between pre-pregnancy BMI and all adverse pregnancy outcomes [13]. Lower pre-pregnancy BMI is associated with an increased risk of maternal anemia. In addition, underweight and greater than normal pre-pregnancy BMI was associated with prolonged preterm rupture of membranes (PPROM), preterm birth, placental abruption, and low birth weight. The authors suggested that optimal pre-pregnancy BMI lay between 19 and 23 kg/m^2^ [14].

In our study, increasing pre-pregnancy BMI was associated with increased cesarean section rates, which was significant even after controlling for traditional risk factors like PCS and birth weight. A study done in Canada showed that a higher BMI was also associated with earlier decisions to perform a cesarean section in the second stage of labor [15]. A recent study in Sweden also revealed that women with a BMI between 25 and 34.9 kg/m^2^ had an increased risk of a cesarean section after labor induction [16]. Obesity is an independent risk factor for cesarean delivery, irrespective of whether it is a primary cesarean section [17]. This implies that the high primary cesarean section rates in the United States may be reduced by optimizing pre-pregnancy BMI.

We found that excessive gestational weight gain, gestational diabetes, hypertensive complications, cesarean delivery, and increased infant weight were higher among women with pre-pregnancy obesity than women with a normal BMI. This was also in keeping with the literature [13] and had implications for preventing pregnancy-related medical conditions before conception.

Interestingly, in the present study, increasing maternal BMI was associated with a decrease in the need for maternal blood transfusion during labor or delivery. A study showed that the association between hemorrhage and BMI might be affected by the mode of delivery. With a vaginal delivery, overweight and obese women had up to 19% increased odds of hemorrhage or atonic hemorrhage. In contrast, women in any obesity class had up to 14% decreased odds of severe hemorrhage after cesarean delivery. The authors concluded that maternal obesity did not have a clinically significant impact on postpartum hemorrhage risk and that the direction of the association between hemorrhage and BMI may differ by mode of delivery [18]. The differences in our study findings may be because we did not adjust for the method of delivery, which may have skewed the results.

We found that the odds of unplanned hysterectomies were higher among overweight mothers, and this increase showed a dose-dependent trend between obesity classes. Baranco et al. found that although the risk of severe maternal morbidity was increased in women with morbid obesity, the rate of unplanned hysterectomy remained unchanged [19]. Our study findings may differ due to our concurrent findings of a reduced need for transfusion with increasing BMI. However, other unmeasured factors may be affecting the decision for an unplanned hysterectomy apart from blood loss, which needs to be explored.

In terms of maternal ICU admissions, underweight and overweight women had no difference in risk compared to women with normal pre-pregnancy BMI. Still, this risk increased across obesity classes, with women in obesity class III having the highest risk of ICU admission. This is in keeping with a study on ICU admission among obstetric patients in whom 82% had a cesarean delivery. Although ICU admission was rare, women with super obesity (defined as BMI > 50kg/m^2^) had the highest risk of ICU admission [20]. This was attributed to the presence of maternal comorbid conditions. In our study, this increased risk of ICU admission remained after adjusting for comorbid conditions indicating an independent and dose-dependent association between BMI and risk of ICU admission.

The present study found a significant association between increasing pre-pregnancy BMI and adverse neonatal outcomes. The risk of having a baby with a low Apgar score (defined as a five-minute Apgar score of less than 7) increases across the BMI classes, with women in obesity class III having the highest risk even after controlling for other risk factors. Furthermore, a systematic review conducted in 2015 revealed that maternal obesity was associated with low Apgar scores at five minutes and large for gestational age neonates, post-term delivery, and stillbirths [21]. This has implications for increased costs and duration of hospital stay for these high-risk newborns who require admission.

The risk of neonates requiring assisted ventilation > six hours increased as their mother's pre-pregnancy BMI increased. The highest risk was seen in women with class III obesity, while underweight mothers showed no increased risk. A similar study showed that infants born to obese level III mothers were much more likely to require assisted ventilation > six hours and NICU admission. These obese mothers also had a higher risk of pregestational and gestational diabetes and cesarean delivery [22]. Even after controlling for these maternal comorbidities in our study, neonatal admission and assisted ventilation risk remained. It is often suggested that obesity-associated inflammation may play a role in adverse neonatal outcomes. Some previous studies demonstrated that increased levels of these inflammatory markers in the neonatal circulation and their direct transmission through the placental barrier could negatively affect the neonate [23].

It has also been shown that maternal obesity is independently associated with an increased incidence of neonatal sepsis and a combination of neonatal morbidities in full-term newborns, including patent ductus arteriosus (PDA), which occurs most commonly in premature babies, and necrotizing enterocolitis [24]. Our observed increased risk for antibiotic use with increasing maternal pre-pregnancy BMI is in tandem with these findings.

Finally, the present study found the highest incidences of neonatal seizures in class II obese women. In general, neonates born to overweight and obese women had a higher incidence of neonatal seizures. For example, a study in Sweden listed maternal obesity among the risk factors for neonatal seizures with an odds ratio of 2.6 for women with a BMI above 40kg/m^2^ [25]. This has significant implications for preventing childhood epilepsy and other neurodevelopmental diseases by optimizing pre-pregnancy BMI.

Study limitations

In the United States Vital Statistics records, the quality of reporting varied widely by state. Multiple data sources, such as discharge summaries and Medicaid claims data, are needed to accurately supplement birth certificate data to understand seizure prevalence in all three US birth settings.

## Conclusions

Though the United States outspends every country in the world on health care, it continues to lag behind the developed world in maternal and child healthcare indices. A significant reason for this may be the astronomical prevalence of obesity in the population and among women of reproductive age. A strong association exists between increasing pre-pregnancy BMI and adverse maternal and neonatal outcomes. The higher risk of adverse pregnancy outcomes among overweight and obese women remained even after controlling for other traditional risk factors of adverse maternal and neonatal outcomes. Since pre-pregnancy BMI is a modifiable risk factor, significant efforts should be directed at obesity prevention, especially among women of reproductive age group, to improve women’s health and pregnancy outcomes.
